# Influence of surface treatment and curing mode of resin composite cements on fibroblast behavior

**DOI:** 10.1186/s13005-022-00323-4

**Published:** 2022-06-11

**Authors:** Nadja Rohr, Celina Baumann, Sabrina Märtin, Nicola U. Zitzmann

**Affiliations:** Biomaterials and Technology, Department of Reconstructive Dentistry, University Center for Dental Medicine Basel UZB, University of Basel, Mattenstrasse 40, CH-4058 Basel, Switzerland

**Keywords:** Cell viability, Fibroblasts, Resin cement, Cytotoxicity test, Light-curing of dental cements

## Abstract

**Background:**

Human gingival fibroblast (HGF-1) cells in the connective tissue provide an effective barrier between the alveolar bone and the oral environment. Cement margins of restorations with intrasulcular preparation or cemented implant restorations are in contact with HGF cells. However, it is unknown to what extend the cement surface finish affects the behavior of HGF cells. The purpose of this study was to compare the behavior of HGF-1 cells in contact with two different resin composite cements with three different surface treatments after light-curing and autopolymerization, respectively.

**Methods:**

Disks of one adhesive (Multilink Automix, Ivoclar Vivadent [MLA]) and one self-adhesive (RelyX Unicem 2 Automix, 3 M [RUN]) resin composite cement were either light-cured or autopolymerized. Specimen surfaces were prepared with the oxygen inhibition layer intact, polished with P2500-grit silicon carbide paper or treated with a scaler. Cells were cultivated on the specimens for 24 h. Viability assay was performed, and cell morphology was examined with scanning electron microscopy. Additionally, roughness parameters of the specimen were analyzed with a 3D laser scanning microscope. Three-way ANOVA was applied to determine the effect of cement material, curing mode and surface treatment (a = 0.05).

**Results:**

Overall, cement material (*p* = 0.031), curing mode (*p* = 0.001), and surface treatment (*p* < 0.001) significantly affected relative cell viability of HGF. The autopolymerized specimen with the oxygen inhibition layer left intact displayed the lowest relative cell viability (MLA 25.7%, RUN 46.6%). Removal of the oxygen inhibition layer with a scaler increased cell viability but also resulted in higher surface roughness values.

**Conclusions:**

HGF cell viability is affected by the surface treatment and the curing mode. The oxygen inhibition layer is an inhibitory factor for the viability of HGF cells. Autopolymerization enhances the cytotoxic potential of the oxygen inhibition layer.

## Background

The soft tissue compartments around teeth and dental implants comprise an epithelial and a connective tissue attachment. In healthy periodontal and peri-implant conditions these attachments provide an effective barrier against penetrating bacteria or bacterial toxins from the oral environment, and protect the underlying alveolar bone [1–3]. Fixed dental prostheses retained on natural abutment teeth, dental implants, or implant abutments, are in contact with the soft tissue compartments. Smooth restoration surfaces are intended to minimize biofilm adherence, ease mechanical biofilm removal, and to facilitate soft tissue attachment. Removal of excess cement is challenging with intrasulcular preparation margins along tooth abutments and with intraoral cementation of implant restorations. A high surface roughness of residual cement promotes biofilm adhesion and subsequently the development of peri-implant diseases [4–8].

Several factors influence the clinicians’ choice of the cement material, such as the restoration material, the abutment preparation design, the retention on the abutment, the position of the restoration margin, and drying possibilities. When using conventional cements such as zinc phosphate or glass ionomer cements, retentive preparations and thin cement margins are required to avoid hydrolysis of exposed cement material. Resin composite cements facilitate adhesion to tooth or implant abutments so that retention is not required and noninvasive preparation designs are feasible [6, 9, 10]. Resin composite cements are characterized by higher strength, lower cement wear, and improved esthetics compared to conventional cements [11–14]. These cements are composed of an organic resin matrix, inorganic and organic filler particles, and silanes [15–17]. The application of an acidic agent and priming system for bonding to tooth structure is required for most adhesive cement systems. Curing of resin composite cements can be catalyzed by either a chemical (autopolymerization), a photo (light-curing) activated initiator, or both (dual-curing), depending on the product. A higher degree of conversion of dual-curing cement is to be expected when light-curing is performed [16, 18, 19]. During radical chain polymerization, the reaction is severely retarded or even stopped by the oxygen in the air causing the formation of a superficial sticky layer on the surface [20, 21]. This so-called oxygen inhibition layer is primarily composed of unreacted monomers, which have been associated with potential cytotoxic effects [22, 23]. Elimination of the oxygen inhibition layer is particularly difficult in the intrasulcular and interdental area where access with hand instruments or rotating polishers is impeded [6], and possibly influences the surrounding soft tissue.

The purpose of this in-vitro study was to evaluate the influence of the curing mode and the removal technique of the oxygen inhibition layer using two different resin composite cements on the viability and morphology of HGF cells. The null-hypotheses were that viability of HGF cells is not affected (i) by light-cured compared to autopolymerized cement surfaces, and (ii) by polished or scaled surfaces compared to specimens with the oxygen inhibition layer left intact.

## Methods

Viability and morphology of human gingival fibroblast (HGF) cells were evaluated after 24 h cultivation on discs of an adhesive (Multilink Automix, Ivoclar Vivadent, Ellwangen, Germany [MLA]) and a self-adhesive resin composite cement (RelyX Unicem 2 Automix, 3 M, Neuss, Germany [RUN]) (Table 1). Cement specimens were produced using either autopolymerization or light-curing and with three different surfaces treatments.

**Table 1:** Cement materials used in this study

Code	MLA	RUN
**Name**	Multilink Automix	RelyX Unicem 2 Automix
**Manufacturer**	Ivoclar Vivadent	3 M
**Type**	Adhesive resin composite cement	Self-adhesive resin composite cement
**Monomers**	Base paste: Bis-GMA, HEMA, 2-dimethylaminoethyl methacrylate Catalyst paste: Ethyoxylated bisphenol A dimethacrylate, UDMA, HEMA	Base paste: TEGDMA, Phosphoric acid- modified methacrylate monomers, Bifunctional methacrylate Catalyst paste: Methacrylate monomers
**Fillers**	40 vol% •Barium glass •Ytterbium trifluoride •Spheroid mixed oxide Particle size: 0.25–3.0 μm	43 vol% •Alkaline (basic) fillers •Silanated fillers Particle size: 12.5 μm
**Initiators**	Dibenzoyl peroxide	Sodium toluene-4- sulphinate, Sodium persulfate, Tert-butyl 3,5,5-trimethylperoxyh- exanoate

Abbreviations: *Bis-GMA* Bisphenol A diglycidylmethacrylate, *HEMA* 2-hydroxyethyl methacrylate, *MLA* Multilink Automix, *RUN* RelyX Unicem 2 Automix, *UDMA* Urethane dimethacrylate, *TEGDMA* Triethylene glycol dimethacrylate

### Production of cement specimens

Discs with a diameter of 13 mm and a thickness of 2 mm were produced using a ring-shaped Teflon mold. The mold was placed on a glass slide, isolated with a Mylar foil, and secured with two clamps. The mold was filled with cement which was smoothed out on the surface using a spatula and either autopolymerized for 1 hour at room temperature, or light-cured for 60 sec from each side with light intensity of 1480 mW/cm^3^ (Elipar DeepCure-S, 3 M Espe, Seefeld, Germany). Discs were not covered to allow the formation of an oxygen inhibition layer. The specimens were placed in an incubator (CTS T^− 4025^; CTS Clima Temperatur Systeme, Hechingen, Germany) for 15 min at 37 °C to allow further polymerization. Discs were removed from the molds and surfaces were either left with the oxygen inhibition layer intact, polished (Minitech 265; Presi, Hagen, Germany) with silica carbide paper grit P2500 (Presi) to simulate the clinical use of a rubber polisher [24], or scraped completely in one direction with a hand scaler to simulate manual cement removal (CLEANext green M23 CN, Deppeler, Rolle, Switzerland) (*n* = 18 per group). Afterwards, specimens were cleaned in an ultrasonic bath with 70% ethanol to avoid contamination for cell experiments. After air-drying, the specimens were stored in sterile 24-well-plates (Falcon; Corning, New York, US).

### Specimen characterization

Surface roughness parameters of the specimens were obtained with a 3D laser scanning microscope (VK-X1050, Keyence, Neu-Isenburg, Germany). Arithmetical mean height Ra and maximum height of profile Rz were measured. For each group, three specimens were analyzed with 11 parallel measurements over a traverse length of 4.8 mm at using a 10x objective (Objective Keyence: Nikon 10x/0.3 WD 16.5 mm). Cut-off filter λ c. = 0.8 μm was applied for all measurements and was chosen according to ISO standard 4288:1996.

### Cell cultivation

Human gingival fibroblasts (HGF-1; ATCC American Type Culture Collection) were cultivated in Dulbecco’s Modified Eagle Medium (DMEM high glucose; Sigma-Aldrich, Darmstadt, Germany). This was supplemented with 1 mL penicillin-streptomycin (Sigma- Aldrich), 1 mL sodium-pyruvate (Gibco; Thermo Fisher Scientific, Waltham, Massachusetts, US), 1 mL L-glutamine (Gibco; Thermo Fisher Scientific), 1 mL amphotericin B solution (Sigma-Aldrich), and 10 mL fetal calf serum (FCS superior; bioswisstec, Schaffhausen, Switzerland), all per 100 mL culture medium. A total of 10^4^ cells (passage 2–3) in 100 μL cell culture medium was placed on each specimen and incubated at 37 °C to attach the cells to the specimen surfaces. Cell experiments were conducted in triplicates with a total of 9 specimens per group per experiment according to the sample size recommendation of ISO standard 10,993-5. Additionally, a blank (specimen with culture medium without cells) for each group of surface treatments was included. Sterile Thermanox coverslips (Thermanox; NUNC, Thermo Fisher Scientific) with a diameter of 13 mm and a thickness of 0.2 mm were used as controls. After 90 min, a further 900 μL cell culture medium was added to each well and cells were cultivated on the specimens for another 22.5 h in the incubator.

### Cell viability assay

A viability assay was conducted after 24 h of direct cell contact with the specimens (cell proliferation reagent WST-1; Sigma-Aldrich). The stable tetrazolium salt WST-1 was converted to soluble formazan by metabolically active cells, resulting in a color change of the solution. Cell culture medium was removed from the wells and 1 mL of WST solution (1:10) was added. The cells were incubated for another 2 h and slightly shaken every 15 min. Afterwards, 3 × 100 μL of solution from each well was transferred to a 96-well-plate and the optical density (OD) was recorded at 490 nm with a microplate reader (RT-2100C Microplate Reader; Versamax, Molecular Devices LLC, San José, California, US). Relative cell viability was calculated using the following equation:

$$\mathrm{Relative}\;\mathrm{cell}\;\mathrm{viability}\;=\;\frac{{\mathrm{OD}}_{\mathrm{specimen}}\;-\;{\mathrm{OD}}_{\mathrm{blank}\;\mathrm{specimen}}}{{\mathrm{OD}}_{\mathrm{control}}\;-\;{\mathrm{OD}}_{\mathrm{blank}\;\mathrm{control}}}$$

### Cell morphology

Scanning electron microscopy (SEM) was used to visualize cell morphology after 24 h cultivation on the specimens (*n* = 3 per group). Cells were rinsed once with PBS, distilled water, and then fixed with glutaraldehyde 2.5% (Sigma Aldrich) for 10 min at room temperature. Afterwards, the specimens were rinsed again with PBS, distilled water, and dehydrated with increasing concentrations of ethanol (30, 50, 70, 80, 90, 96%, abs.) changed every 15 min. Specimens were then dried in a desiccator with silica gel and sputtered with gold. SEM analysis was performed at 15 kV, 2000x magnification (ESEM XL-30, Philips, SEMTech Solutions, North Billerica, Massachusetts, US).

### Statistical analysis

Relative cell viability and surface roughness values were tested for normal distribution using Shapiro-Wilk test. Three-way ANOVA was applied to determine the effect of cement material, curing mode and surface treatment. Post-hoc Fisher’s least significant difference test was conducted for differences within subgroups. The level of significance was set to a = 0.05 (StatPlus Pro V6.1.25, AnalystSoft, Alexandria, Virginia, US).

## Results

### Specimen characterization

The roughness parameters of all specimens are presented in Table 2. Ra values were significantly affected by the cement material (*p* = 0.019), the curing mode (*p* < 0.001), and the surface treatment (*p* < 0.001) with three-way ANOVA. 2). A significant effect for all three tested factors was also found for Rz values with ANOVA (all *p* < 0.001).

**Table 2:** Surface roughness (μm) parameters of specimens of resin composite cements MLA (Multilink Automix) and RUN (RelyX Unicem 2 Automix) with means and standard deviation of arithmetical mean height (R_a_) and maximum height of profile (R_z_)

		Ra		Rz	
**Curing mode**	**Surface treatment**	**MLA**	**RUN**	**MLA**	**RUN**
Autopolymerization	Oxygen inhibition layer	1.82 ± 0.20^Aa^	3.94 ± 1.21^Ab^	12.90 ± 1.80^Aa^	32.00 ± 12.05^Ab^
	Polished	0.71 ± 0.04^Ba^	0.65 ± 0.03^Ba^	5.17 ± 0.31^Ba^	4.92 ± 0.33^Ba^
	Scaler	3.12 ± 0.42^Ca^	2.15 ± 0.84^Cb^	18.02 ± 1.62^Ca^	16.19 ± 7.96^Cb^
Light-curing	Oxygen inhibition layer	0.96 ± 0.14^Da^	0.83 ± 0.08^Bb^	8.23 ± 1.95^Da^	7.20 ± 1.26^BDb^
	Polished	0.67 ± 0.06^Ba^	0.64 ± 0.07^Ba^	4.65 ± 0.55^Ba^	4.77 ± 0.57^Ba^
	Scaler	2.84 ± 0.46^Ea^	1.25 ± 0.13^Cb^	15.41 ± 1.83^Ea^	8.55 ± 0.73^Db^

Statistical differences between groups of Ra and Rz respectively determined with Fisher’s LSD post-hoc test are indicated with differing superscript letters (uppercase vertical comparison, lowercase horizontal comparison)

**Fig. 1:**
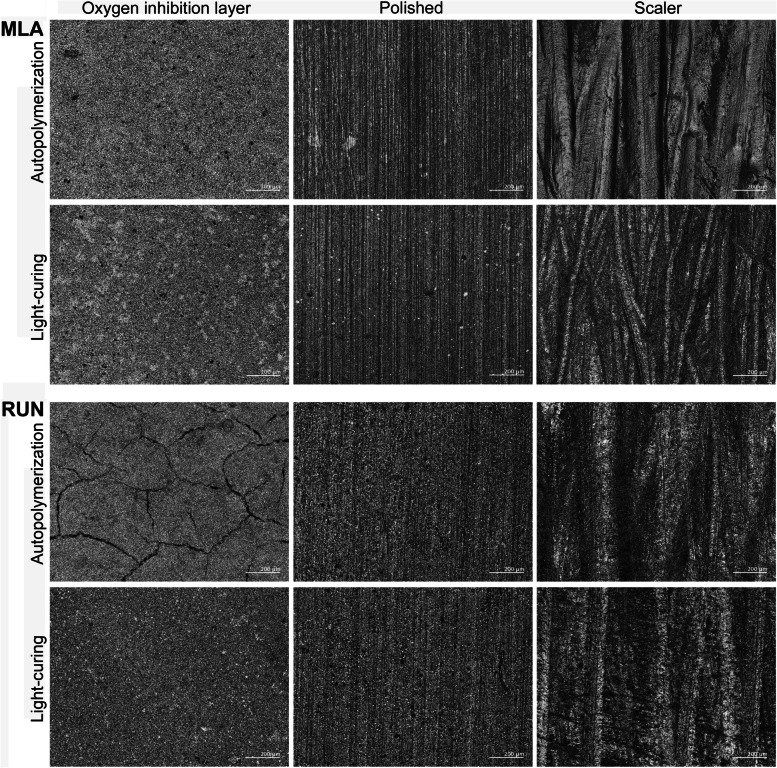
3D laser scanning microscope images of resin composite cement specimens. Scale bar is 200 μm. MLA, Multilink Automix. RUN, Rely X Unicem

Images obtained with the 3D laser scanning microscope revealed that RUN specimens displayed a surface with irregular wider cracks when the specimens were autopolymerized (Fig. 1). Autopolymerization generally resulted in significantly higher Ra values than light-cured polymerization, especially for specimens left with the oxygen inhibition layer intact (*p* < 0.001) and those treated with a scaler (*p* < 0.001), while no difference between curing modes was observed for polished specimens (*p* = 0.928). Using the scaler, the oxygen inhibition layer was partially removed, but increased the surface roughness parameters, except for autopolymerized specimens of RUN treated with the scaler which had significantly lower Ra values compared to the untreated autopolymerized RUN specimens (*p* < 0.001).

### Cell viability

Mean values and standard deviation of the relative cell viability after 24 h are presented in Table 3. Three-way ANOVA revealed that cement material (*p* = 0.031), curing mode (*p* = 0.001), and surface treatment (*p* < 0.001) significantly affected relative cell viability of HGF. The mean relative cell viability ranged from 25.7 ± 20.2% for autopolymerized MLA specimens left with the oxygen inhibition layer intact to 111.9 ± 31.4% for light-cured RUN specimens treated with a scaler. For both cements, the relative cell viability was lowest for autopolymerized specimens with the oxygen inhibition layer left intact (*p* < 0.001). No correlation was observed between surface roughness and relative cell viability values of the respective groups.

**Table 3:** Relative cell viability (%) mean and standard deviations of HGF cells on resin composite cements MLA (Multilink Automix) and RUN (RelyX Unicem 2 Automix)

Curing mode	Surface treatment	MLA	RUN
Autopolymerization	Oxygen inhibition layer	25.7 ± 20.2^Aa^	46.6 ± 13.3^Aa^
	Polished	86.0 ± 22.4^BCa^	85.6 ± 12.9^BCa^
	Scaler	88.2 ± 32.7^BCa^	75.0 ± 16.9^Ba^
Light-curing	Oxygen inhibition layer	68.7 ± 26.5^Ba^	93.2 ± 25.2^BDb^
	Polished	87.8 ± 11.8^BCa^	110.0 ± 23.2^CDa^
	Scaler	105.0 ± 18.6^Ca^	111.9 ± 31.4^Da^

Statistical differences between groups determined with Fisher’s LSD post-hoc are indicated with differing superscript letters (uppercase vertical comparison, lowercase horizontal comparison)

### Cell morphology

**Fig. 2:**
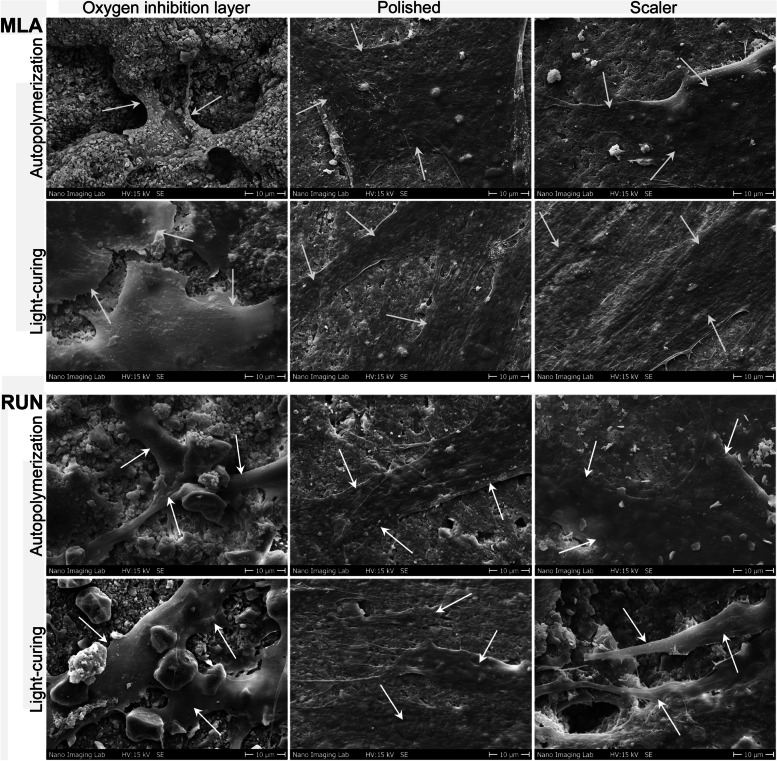
Scanning electron microscopic images of resin composite cement specimen surfaces. Scale Bar is 10 μm. White arrows indicate HGF cells. MLA, Multilink Automix. RUN, Rely X Unicem

SEM images of the specimens are presented in Fig. 2. Differences in fibroblast morphology were observed between the types of curing mode and surface treatments. On light-cured and autopolymerized specimens that were either polished or treated with a scaler, cell spread without a specific orientation but with flat and tight attachment to the surfaces was observed, while filopodia spread less on surfaces with the oxygen inhibition layer. Some cell residuals could be detected on autopolymerized MLA specimens that were not surface treated. Cells on autopolymerized RUN specimens with the oxygen inhibition layer showed a different, spherical morphology. Filopodia were formed by cells on all surfaces, but most noticeably on light-cured specimens.

## Discussion

This in-vitro study investigated the impact of the curing mode and surface treatment on HGF cell viability and morphology for two resin composite cements. The results showed that the cell viability was mainly affected by the curing mode but also the surface treatment of the cement. Higher cell viability values were measured for light-cured specimens compared to autopolymerized specimens, and for polished or scaled surfaces compared to surfaces with the oxygen inhibition layer left intact. Both hypotheses were rejected and supported by matching SEM images which showed that the morphology of HGF cells was influenced by the curing mode and surface treatment.

The surface treatment protocols in this study were selected to reflect those encountered in clinical situations, comprising no treatment due to insufficient access to cement margins resulting in the oxygen inhibition layer remaining, polishing of excess cement using a rubber polisher [24], and cement removal with a scaler. The impact of the application of a glycerin paste to prevent oxygen inhibition layer formation was considered in a pre-test, but was not pursued due to highly uneven surfaces with great variability and an unpredictable outcome. As the specimens were rinsed with ethanol prior to cell experiments to limit the risk of contamination, cytotoxic components such as monomers may have been partially removed prior to the cell experiments. Therefore, the cytotoxic effect may be even higher in a clinical situation [22].

Further, cell activity and cell proliferation, which affect cell viability assays are very complex metabolic processes, and in-vitro data cannot be directly transferred to the clinical setting.

Previously, it has been shown that cell viability was significantly higher on smooth cement surfaces with Ra values between 0.2 and 0.8 μm [6]. These results are not confirmed by the findings of this study where polished and scaler-treated surfaces displayed strong variations in surface roughness values, but this variation in roughness parameters did not seem to affect the cell viability. Therefore, it can be assumed that the impact of the different surface treatments (or leaching components) on the cell viability was stronger than the impact of the surface roughness. Interestingly, the microscopic imaging revealed that light-curing can apparently generate smoother surfaces compared with autopolymerized surfaces. Autopolymerized surfaces of RUN displayed cracks while surfaces of MLA appeared smooth. Those cracks may have occurred during production when the samples were moved to 37 °C after being stored for 60 minutes at room temperature. Rising temperature during storage from 23 °C to 37 °C significantly increased the indirect tensile strength of RUN but not MLA, which indicates that the polymerization process of RUN is strongly affected by temperature [25].

Surfaces that were treated with the scaler appeared rougher when autopolymerized and higher Rz values were measured for both cement materials than for light-cured specimens, which may indicate that the material remains softer on the surface due to insufficient polymerization and the scaler treatment thus leads to deeper scratches on the surface. The thickness of the formed oxygen inhibition layer on the surface of light-cured compared to autopolymerized specimens should be further analyzed and hardness measurements may be performed.

Rohr et al. [6] found that RUN contains silica particles and aluminum fluoride fillers with a filler size of 12.5 μm. MLA showed a filler size of 0.25–3.0 μm and additionally, zirconia fillers, ytterbium trifluoride, and barium glass were added. With the energy dispersive X-ray analysis analyses of previous studies, only the inorganic components, such as the fillers, could be analyzed. In none of the six tested resin composite cements did the cement type or composition correlate with cell viability [6]. In addition, the biocompatibility did not seem to be affected by the fillers of resin composite material [26]. An in-vitro study compared the cytotoxic effects of different types of monomers and observed the following range of increased toxicity: hydroxyethylenethylmethacrylate (HEMA) < triethyleneglycoldimethacrylate (TEGDMA) < urethanedimethacrylate (UDMA) < bisglycidylmethacrylate (Bis-GMA). The increase in toxicity was proportional to the increase in molecular mass [23]. RUN is composed of TEGDMA and other methacrylates [27, 28], but the manufacturers did not specify the quantity of monomers the cement contains [27]. As described in MLA’s safety data sheets, the base paste contains 10–25% Bis-GMA and ≥ 2.5 to < 10% HEMA [29]. The catalyst paste contains 2.5 to < 10% UDMA and 2.5 to < 10% HEMA [30]. The cements analyzed in the present study contained different monomers, which could possibly explain the significant difference in cell viability between both cements when not sufficiently polymerized for the specimens left with the oxygen inhibition layer intact.

In the present study, higher viability was observed for cells on light-cured specimens compared to autopolymerized specimens. Hence, the potentially higher degree of polymerization with light-curing was found to have a more severe impact on cell viability than the surface roughness. Dual-cured cements generally reached a higher degree of conversion when light-curing was performed [16, 18, 19].

Previous studies have shown that monomer release had a toxic effect on L^− 929^ mouse fibroblasts, especially if cements were not sufficiently polymerized [31, 32]. Additionally, a reduction of curing time significantly promoted the cytotoxicity of methacrylate-based materials [31]. Although neither the degree of conversion nor the amount of monomer leaking from the surface have been quantified, it can be assumed that light-cured specimens displayed a higher degree of conversion than autopolymerized specimens. Additionally, the presence of residual monomers on the untreated specimens with the oxygen inhibition layer was probably higher than that on the surfaces treated subtractively by polishing or with the scaler. The relative cell viability values for autopolymerized specimens left with the oxygen inhibition layer intact (RUN 46.6 ± 13.3%, MLA 25.7 ± 20.2%) are < 70% and have to be interpreted as cytotoxic effects according to ISO standard 10,993-5 [33]. However, when the surface of resin composite cements is light-cured and the oxygen inhibition layer is manually removed, a cell viability of fibroblasts even higher than on the control surface polystyrene can be expected.

## Conclusions

In conclusion, the oxygen inhibition layer is an inhibitory factor for the viability of fibroblasts; hence if accessible, the surface of the cement margins should be finished with a rubber polisher or scaler to remove the superficial layer, which is probably not sufficiently polymerized. Clinicians should perform light-curing of the cement margin of a restoration to increase fibroblast viability.

## Data Availability

The datasets used and analyzed during the current study are available from the corresponding author on reasonable request.
